# Poricoic acid A suppresses renal fibroblast activation and interstitial fibrosis in UUO rats via upregulating Sirt3 and promoting β-catenin K49 deacetylation

**DOI:** 10.1038/s41401-022-01026-x

**Published:** 2022-12-05

**Authors:** Dan-Qian Chen, Lin Chen, Yan Guo, Xia-Qing Wu, Ting-Ting Zhao, Hai-Ling Zhao, Hao-Jun Zhang, Mei-Hua Yan, Guo-Qiang Zhang, Ping Li

**Affiliations:** 1 Department of Emergency, China-Japan Friendship Hospital, Beijing, 100029 China; 2 Faculty of Life Science & Medicine, Northwest University, Xi’an, 710069 China; 3 Department of Internal Medicine, University of New Mexico, Albuquerque, NM 87131 USA; 4 Beijing Key Lab for Immune-Mediated Inflammatory Diseases, Institute of Clinical Medical Sciences, China-Japan Friendship Hospital, Beijing, 100029 China

**Keywords:** renal interstitial fibrosis, sirt3, poricoic acid A, β-catenin, deacetylation, fibroblast activation, unilateral ureteral obstruction (UUO)

## Abstract

Renal interstitial fibrosis is the common pathological process of various chronic kidney diseases to end-stage renal disease. Inhibition of fibroblast activation attenuates renal interstitial fibrosis. Our previous studies show that poricoic acid A (PAA) isolated from *Poria cocos* is a potent anti-fibrotic agent. In the present study we investigated the effects of PAA on renal fibroblast activation and interstitial fibrosis and the underlying mechanisms. Renal interstitial fibrosis was induced in rats or mice by unilateral ureteral obstruction (UUO). UUO rats were administered PAA (10 mg·kg^−1^·d^−1^, i.g.) for 1 or 2 weeks. An in vitro model of renal fibrosis was established in normal renal kidney fibroblasts (NRK-49F cells) treated with TGF-β1. We showed that PAA treatment rescued Sirt3 expression, and significantly attenuated renal fibroblast activation and interstitial fibrosis in both the in vivo and in vitro models. In TGF-β1-treated NRK-49F cells, we demonstrated that Sirt3 deacetylated β-catenin (a key transcription factor of fibroblast activation) and then accelerated its ubiquitin-dependent degradation, thus suppressing the protein expression and promoter activity of pro-fibrotic downstream target genes (twist, snail1, MMP-7 and PAI-1) to alleviate fibroblast activation; the lysine-49 (K49) of β-catenin was responsible for Sirt3-mediated β-catenin deacetylation. In molecular docking analysis, we found the potential interaction of Sirt3 and PAA. In both in vivo and in vitro models, pharmacological activation of Sirt3 by PAA significantly suppressed renal fibroblast activation via facilitating β-catenin K49 deacetylation. In UUO mice and NRK-49F cells, Sirt3 overexpression enhanced the anti-fibrotic effect of PAA, whereas Sirt3 knockdown weakened the effect. Taken together, PAA attenuates renal fibroblast activation and interstitial fibrosis by upregulating Sirt3 and inducing β-catenin K49 deacetylation, highlighting Sirt3 functions as a promising therapeutic target of renal fibroblast activation and interstitial fibrosis.

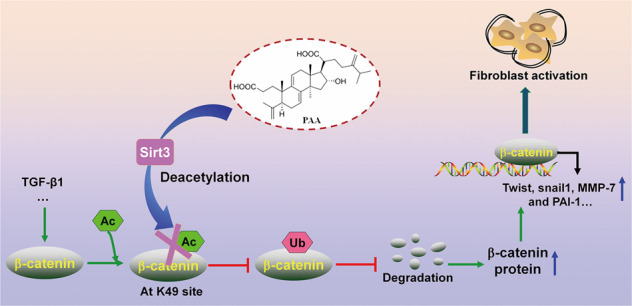

## Introduction

Chronic kidney disease (CKD) has high morbidity and mortality and cannot be thoroughly healed [1]. Approximately 10% of adults have suffered CKD, and the global burden has been rapidly growing [2]. Renal interstitial fibrosis is the final and common pathological process from various CKDs to end-stage renal disease [3]. During renal interstitial fibrosis process, fibroblast is the main effect cell and fibroblast activation is the main characteristics. Fibroblast is normally quiescent and sporadically dispersed in kidney, while they are activated and mitigate to injured sites after chronic inflammation [4]. Activated fibroblasts in kidney secrete massive chemokines and growth factors, resulting in decreased E-cadherin and increased vimentin and α-SMA, and eventually evoke renal interstitial fibrosis and kidney function decline [5]. In this context, it is conceivable to speculate that the inhibition of fibroblast activation is the promising therapeutic strategy to delay renal interstitial fibrosis and CKD.

A number of transcription factors regulate renal fibroblast activation and interstitial fibrosis [6]. One important transcription factor is β-catenin [7]. β-Catenin is silent in normal situation, but becomes activated once kidney is injured. Excessive β-catenin accumulate in the cytoplasm and translocate into the nucleus, then initiate the expression of pro-fibrotic downstream target genes, including twist, snail1, matrix metalloproteinase-7 (MMP-7), and plasminogen activator inhibitor-1 (PAI-1) [8]. The stability of β-catenin promotes renal fibroblast activation and interstitial fibrosis, while its instability alleviates fibrosis via reducing the expression of downstream target genes [9]. Previous studies indicate that posttranslational modification of β-catenin at the N-terminal 1–49 amino acids determines its stability including phosphorylation and ubiquitination, and notably, the acetylation of β-catenin protein stabilizes β-catenin [10, 11], which might be a potential target to inhibit β-catenin activity and then alleviate renal fibroblast activation and interstitial fibrosis. The acetylation occurs at lysine residues, and lysine residues in β-catenin are lysine-11, lysine-19 (K19), lysine-49 (K49), and lysine-394. Considering that the importance of N-terminal 1–49 amino acids in maintaining β-catenin stability [9], we mainly focus on K19 and K49 sites in this study.

Sirt3 is a member of mammalian sirtuins that is an evolutionarily conserved family of nicotinamide adenine dinucleotide (NAD^+^)-dependent deacetylases. The pleotropic identity of Sirt3 is manifested into several catalytic activities to influence the biological function of various cell types and tissues. Sirt3 alleviates fibrosis progression in multiple organs [12–14]. In our previous study, we found that Sirt3 could affect Wnt/β-catenin pathway activity mainly via modulating Wnt ligands [15], which is consistent with other studies [16, 17]. But the direct regulatory effect of Sirt3 on β-catenin deacetylation remains obscure, and no evidence supports that Sirt3 could mediate β-catenin deacetylation. Here, one of our efforts is investigating the effect and underlying mechanism of Sirt3 on β-catenin deacetylation during renal fibroblast activation and interstitial fibrosis.

Natural product is the crucial and irreplaceable source to seek promising drug against renal fibroblast activation and interstitial fibrosis [8]. *Poria cocos* (Schw.) Wolf (Poliporaceae) is an edible and medicinal mushroom, and the sclerotia is also known as “tuckahoes” or “Indian bread”. Our previous studies prove the protection of *Poria cocos* against renal fibrosis [18, 19], and poricoic acid A (PAA) is the main active component. PAA, a tetracyclic triterpenoid component isolated from *Poria cocos*, significantly reduces renal fibrosis and prevents declined kidney function [20, 21]. Although PAA ameliorates inflammation and abnormal extracellular matrix (ECM) remodeling to prevent renal fibrosis and CKD progression [20, 22, 23], the effect and underlying mechanism of PAA on fibroblast activation remain unclear. Here, another effort is aimed to elucidate the inhibitory effect of PAA on renal fibroblast activation and underlying mechanism.

In this study, we comprehensively explored the inhibitory effect and mechanism of PAA on renal fibroblast activation and interstitial fibrosis. Our data revealed that Sirt3 was required for PAA to attenuate renal fibroblast activation and interstitial fibrosis, and firstly demonstrated that Sirt3 deacetylated β-catenin mainly at K49 site thus suppressing β-catenin stability and following pro-fibrotic downstream target gene expressions, highlighting Sirt3 functioned as a promising therapeutic target of renal fibrosis.

## Materials and methods

### Animal treatment and study approval

All animal care and experimental procedures were approved by the Ethics Committee of the Institute of Chinese Materia Medica, China Academy of Chinese Medical Sciences (No. 20152013). All animal care and experimental procedures were performed in strict accordance with the Guide for the Care and Use of Laboratory Animals of the State Committee of Science and Technology of the People’s Republic of China. Animal studies are reported in compliance with the ARRIVE guidelines [24]. Animals were kept under specific pathogen-free conditions at constant temperature and humidity, with a 12-h light cycle, and were provided with food and water ad libitum. Male Sprague-Dawley rats weighing 180–200 g were purchased from the Laboratory Animal Center of the Academy of Military Medical Sciences (Beijing, China, Certificate No. SCXK 2002-0010) and used to establish unilateral ureteral obstruction (UUO) model that was carried out as described previously [25]. Male Sirt3^−/−^ C57BL/6 mice weighing 20–22 g were provided by Cyagen (Suzhou, China; https://www.cyagen.com/cn/zh-cn/), and control littermates were used as wild type (WT).

**Fig. 1:**
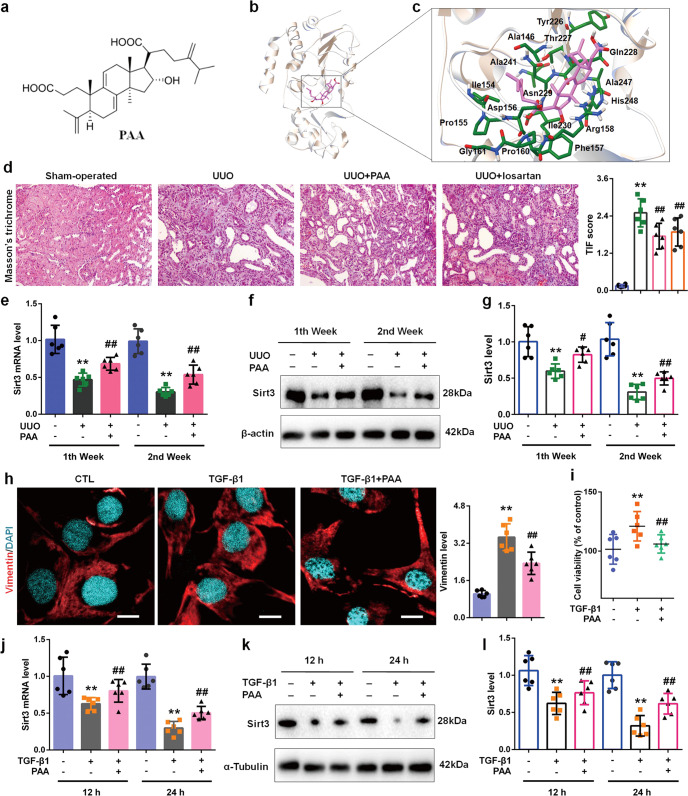
PAA activates Sirt3 during renal fibroblast activation and interstitial fibrosis. **a** The chemical structure of PAA. **b** Binding models of PAA (pink) in Sirt3 (white). **c** The interaction of PAA with the amino acid of Sirt3. **d** Masson’s trichrome staining of kidney tissues of UUO rats at 2nd week. Magnification, ×100. **e** The mRNA expression of Sirt3 in UUO rats. **f**, **g** The protein expression and relative quantitative data of Sirt3 in UUO rats. **h** Immunofluorescent staining of vimentin and relative quantitative data in NRK-49F cells after 24 h treatment. Scale bar, 50 μm. **i** The cell viability of NRK-49F cells after 24 h treatment. **j** The mRNA expression of Sirt3 in NRK-49F cells. **k**, **l** The protein expression and relative quantitative data of Sirt3 in NRK-49F cells. Data were presented as mean ± SD. Each dot presented the single data result in bar graph. ^**^P < 0.01 *vs* sham-operated or control group (*n* = 6). ^*#*^*P* < 0.05, ^##^*P* < 0.01 vs UUO or TGF-β1 group (*n* = 6)

PAA was purchased from Topscience (T8181, Shanghai, China) and given to mice or rats at the dose of 10 mg/kg by intragastric administration each day. The chemical structure of PAA is shown in Fig. 1a. Losartan was purchased from Topscience (T0215L, Shanghai, China) and used as positive drug at the dose of 30 mg/kg per day by intragastric administration. Rats were randomized into three groups: sham-operated group (*n* = 8), UUO group (*n* = 8), UUO + PAA group (*n* = 8), and UUO + losartan group (*n* = 8), and sacrificed at 1st and 2nd weeks respectively. WT and Sirt3^-/-^ mice were randomized into three groups: sham-operated group (*n* = 8), UUO group (*n* = 8), and UUO + PAA (*n* = 8), and sacrificed at the 1st week. The ligated kidneys were immediately frozen by liquid nitrogen.

### Cell culture and treatment

Normal renal kidney fibroblasts (NRK-49F) were obtained from the China Center for Type Culture Collection (Beijing, China) and cultured at 37 °C with 5% CO_2_ in DMEM/F-12 with 10% tst-system approved fetal bovine serum. The 2.5 ng/mL recombinant human TGF-β1 protein (7754-BH, R&D system, Emeryville, CA, USA) was used to induce fibroblast activation, and 10 μM PAA was used to activate Sirt3 activity. The 50 μg/mL cycloheximide (HY-12320, MedChemExpress, Shanghai, China) was used to block protein synthesis. After 12 h or 24 h treatment, NRK-49F cells were harvested for subsequent experiments.

### Plasmid construction and transfection in vitro

Lentivirus expressing full-length rat Sirt3 cDNA (Sirt3 over), lentivirus containing empty plasmids (vector), lentivirus expressing shRNA against rat Sirt3 (Sirt3 shRNA), lentivirus expressing scramble (scramble), and lentivirus expressing full-length rat β-catenin cDNA (β-catenin WT) were constructed by Genechem (Shanghai, China). Mutation of β-catenin at K19 and K49 into arginine (K19R, K49R) or glutamine (K49Q) were also constructed by Genechem (Shanghai, China). When NRK-49F cells have reached 70%–80% confluence, the transfection complexes were added into the medium and the medium was removed after 8 h treatment. The expression of Sirt3 protein after transfection with lentivirus was shown in Supplementary Fig. S1.

### Western blot

The protocol of Western blot has been described previously [21]. Briefly, kidney tissues and NRK-49F cells were extracted using RIPA lysis buffer (89901, Thermo Scientific, Waltham, MA, USA) and the protein concentration was detected by BCA protein assay kit (23227, Thermo Scientific, Waltham, MA, USA). After 10%–20% SDS-PAGE electrophoresis, the proteins were transferred onto the polyvinylidene difluoride membranes (10600023, GE Healthcare, Boston, MA, USA) and blocked using 5% BSA buffer at room temperature for 1 h. The membranes were incubated with primary antibodies (listed in Supplementary Table S1) at 4 °C overnight and then incubated with secondary antibodies at room temperature for 1 h. The immunoreactive bands were visualized using chemiluminescence Western blotting detection reagent (32209, Thermo Scientific, Waltham, MA, USA) and the densitometry was performed by using ImageJ software (v1.48, NIH, Bethesda, MD, USA).

### Co-immunoprecipitation (Co-IP)

Co-IP was performed using Dynabeads^TM^ Protein A Immunoprecipitation Kit (10006D, Thermo Scientific, Waltham, MA, USA) as the following method routinely employed in our lab [22]. Briefly, the lysates of kidney tissues or NRK-49F cells were pretreated with Protein A for 1 h at 4 °C, and then the supernatant was incubated with anti-β-catenin (1:100) at 4 °C overnight. The supernatant was immunoprecipitated by protein A at 4 °C overnight to obtain the immune complexes, and the immunoprecipitants were analyzed using Western blotting.

### Quantitative real-time polymerase chain reaction (qRT-PCR)

qRT-PCR was performed as the following method routinely employed in our lab [22]. Total RNA was extracted using TRIzol reagent (T9108, Takara Bio, Dalian, China). The cDNA was synthesized using Transcriptor First Strand cDNA Synthesis Kit (Roche, Basel, Switzerland) and qRT-PCR was performed using SYBR^®^ Premix Ex Taq^TM^ II (Takara Bio, Dalian, China) in an Applied Biosystems 7500 system (Thermo Scientific, Waltham, MA, USA). The experiments were carried out in triplicate, and the gene expression levels were normalized to β-actin using the 2^−ΔΔCt^. The primers’ sequences were listed in Supplementary Table S2.

### Immunofluorescence staining and confocal microscopy

The cultured NRK-49F cells were fixed in 4% paraformaldehyde at 4 °C for 15 min and then incubated with anti-vimentin (1:200) at 4 °C overnight. The secondary antibodies conjugated to Alexa Fluor 488/594 (Abcam, Cambridge, MA, USA) were added to the slides at room temperature for 30 min, and DAPI was used to stain the nucleus. The image was obtained using a laser confocal microscope (Zeiss, Oberkochen, Germany).

### Cell viability

Cell viability was measured using CCK-8 kit (C0037, Beyotime, Shanghai, China). NRK-49F cells (1 × 10^5^) in 96-well plates were treated with TGF-β1 and/or PAA for 24 h, then CCK-8 was added into the medium and incubated for 30 min. After washing with PBS, the absorbance was measured at 450 nm.

### Chromatin immunoprecipitation (ChIP)

ChIP was performed as the following method routinely employed in our lab [22]. ChIP assay was performed using Pierce Agarose ChIP Kit (26156, Thermo Scientific, Waltham, MA, USA) according to the manufacturer’s instructions. NRK-49F cells were incubated with 1% formaldehyde for 10 min to crosslink DNA/protein complexes, then the chromatin was digested using micrococcal nuclease at 4 °C for 5 min. Then, the lysates were incubated with anti-β-catenin antibody (1:100) or normal rabbit IgG as a negative control at 4 °C overnight. The promoter activity of downstream target genes was measured using qRT-PCR method. The primers’ sequences were listed in Supplementary Table S2.

### Histological analysis

Haematoxylin and Eosin (H&E) and Masson’s trichrome staining of kidney tissues and quantitative analysis were performed as described previously [21, 23].

### T-cell factor/lymphoid enhancer-binding factor (TCF/LEF) reporter assay

TCF/LEF reporter assay was performed using Cignal TCF/LEF reporter assay kit (336841, Shanghai, China) according to the manufacturer’s protocol. Briefly, NRK-49F cells were transfected with the p(GAGA)12-luc and pGL3-basic using Lipofectamine 3000 (Invitrogen, Carlsbad, CA, USA). The fluorescence was measured using the dual-luciferase reporter assay system (E1910, Promega, Madison, WI, USA).

### Human subjects and study approvals

The clinical study was approved by the Ethical Committee of Clinical Center, Shaanxi Traditional Chinese Medicine Hospital (SXSY-235610). The clinical investigation has been performed on the basis of the principles expressed in the Declaration of Helsinki. The informed consents were acquired from participants prior to inclusion in this study. The detailed information of human subjects was consistent with previous study [23].

### Molecular docking

Molecular docking was performed using AutoDock 4.2 as previously described [18]. The crystal structure of human Sirt3 was acquired from Protein Data Bank. The PAA minimization was performed with MOE before analysis. The grid boxes of Sirt3 were set at 90 Å × 120 Å × 100 Å points, and a grid spacing of 0.375 Å was used. Discovery Studio 4.5 Visualizer was used to analyze the interaction between PAA and amino acids of Sirt3.

### Sirt3 deacetylase activity assay

Sirt3 deacetylase activity was determined by using SIRT3 Activity Assay Kit (ab156067, Abcam, Cambridge, MA, USA) according to the manufacturer’s instruction. NRK-49F cells were treated with TGF-β1 and/or PAA for 12 h and 24 h. Sirt3 activity was measured by detecting fluorescent emission at 460 nm and following excitation at 360 nm.

### Statistics analysis

The results were presented as mean ± SD, and each data set contains 6 replicates per group unless stated otherwise. Data were analyzed using commercial software (GraphPad 6, San Diego, CA, USA). Statistical significance was calculated using a two-tailed unpaired Student’s *t* test between two groups, and using one-way analysis of variance followed by Dunnett’s post hoc test among multiple comparisons. Values of *P* < 0.05 were considered statistically significant.

## Results

### PAA prevents Sirt3 downregulation during renal fibroblast activation and interstitial fibrosis

To prove the regulatory effect of PAA on Sirt3, we used molecular docking analysis to observe the possible interaction of Sirt3 and PAA, and found their potential interaction (Fig. 1b, c). Further experiments explored the regulatory effect of PAA on Sirt3 expression and underlying mechanism by using UUO model. As shown in Fig. 1d, Masson’s trichrome staining results showed that severe tubular dilation and massive collagen deposition on renal interstitium were observed after UUO surgery at 2nd week compared to sham-operated group, while treatment with PAA or losartan significantly alleviated collagen deposition and tubular dilation. As shown in Fig. 1e–g, compared with sham-operated group, Sirt3 mRNA and protein expression decreased in UUO group at 1st and 2nd weeks, while PAA treatment rescued Sirt3 mRNA and protein expression after UUO surgery.

Similar results were observed in cultured NRK-49F cells. TGF-β1 was used to induce fibroblast activation. Immunofluorescence staining results showed that vimentin (a maker of fibroblast activation) was increased after TGF-β1 stimulation, but decreased by PAA treatment (Fig. 1h). Cell viability results also indicated that PAA treatment obviously inhibited fibroblast viability and activation (Fig. 1i). As shown in Fig. 1j-l, TGF-β1 stimulation caused the downregulation of Sirt3 mRNA and protein in NRK-49F cells, while PAA treatment upregulated Sirt3 mRNA and protein expression after 12 h and 24 h treatment. Notably, PAA also contributed to Sirt3 deacetylase activity in NRK-49F cells after 12 h and 24 h treatment (Supplementary Fig. S2). These evidences supported that PAA rescued Sirt3 expression and activity during renal fibroblast activation and interstitial fibrosis, indicating the pharmacological activation of Sirt3 by PAA.

### Sirt3 suppresses renal fibroblast activation and interstitial fibrosis via mediating β-catenin deacetylation

**Fig. 2:**
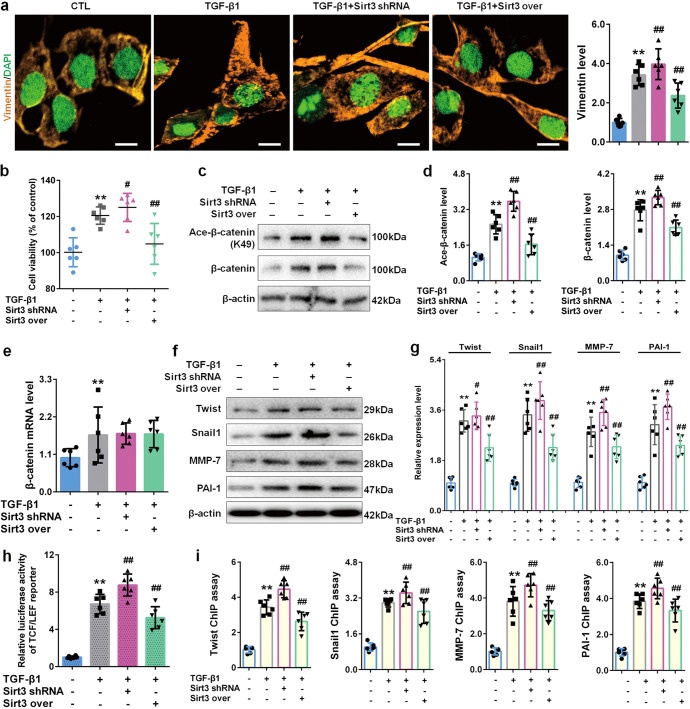
Sirt3 exerts the inhibitory effects on renal fibroblast activation through deacetylating β-catenin. **a** Immunofluorescent staining of vimentin and relative quantitative data in NRK-49F cells after 48 h treatment. Scale bar, 50 μm. **b** The cell viability of NRK-49F cells after 48 h treatment. **c**, **d** The protein expression and relative quantitative data of ace-β-catenin (K49) and β-catenin in NRK-49F cells after 48 h treatment. **e** The mRNA expression of β-catenin in NRK-49F cells after 48 h treatment. **f**, **g** The protein expression and relative quantitative data of twist, snail1, MMP-7 and PAI-1 in NRK-49F cells after 48 h treatment. **h** The luciferase activity of TCF/LEF reporter in NRK-49F cells after 48 h treatment. **i** ChIP assay results of snail1, twist, MMP-7, and PAI-1 in NRK-49F cells after 48 h treatment. Data were presented as mean ± SD. Each dot presented the single data result in bar graph. ^**^P < 0.01 vs control group (*n* = 6). ^#^*P* < 0.05, ^##^*P* < 0.01 vs TGF-β1 group (*n* = 6)

Further experiments investigated the anti-fibrosis of Sirt3 and its underlying mechanism in kidney. Firstly, we used lentivirus expressing Sirt3 over and Sirt3 shRNA to verify its anti-fibrotic effect. As shown in Fig. 2a, immunofluorescence staining of vimentin (a marker of fibroblast activation) results revealed that Sirt3 deficiency intensified TGF-β1-induced vimentin upregulation, while Sirt3 overexpression significantly lowered vimentin upregulation. Cell viability results showed that Sirt3 deficiency contributed to fibroblast viability, while Sirt3 overexpression suppressed fibroblast viability in NRK-49F cells (Fig. 2b). These data confirmed the effect of Sirt3 against fibroblast activation in kidney.

To fully address that the regulatory effect of Sirt3 on β-catenin, we measured β-catenin and its pro-fibrotic downstream target gene expression at different levels. As shown in Fig. 2c, d, TGF-β1 stimulation caused the upregulation of acetylated β-catenin and total β-catenin in NRK-49F cells. Notably, Sirt3 deficiency potentiated the upregulation of acetylated β-catenin and total β-catenin protein, while Sirt3 overexpression significantly suppressed the upregulation of acetylated β-catenin and total β-catenin protein. As shown in Fig. 2e, TGF-β1 stimulation increased the mRNA level of β-catenin in NRK-49F cells, but neither Sirt3 shRNA nor Sirt3 over hardly affected β-catenin mRNA level. These evidences suggested that Sirt3 only affected β-catenin expression and deacetylation modification at protein level rather than at mRNA level.

β-Catenin contributes to fibroblast activation and fibrosis mainly through promoting its pro-fibrotic downstream target gene expression, especially twist, snail1, MMP-7 and PAI-1 [7]. Next, we determined the expression of pro-fibrotic downstream target genes at different levels. As shown in Fig. 2f, g, Sirt3 deficiency intensified the upregulation of twist, snail1, MMP-7 and PAI-1, while Sirt3 overexpression significantly restrained the upregulation of twist, snail1, MMP-7 and PAI-1. Sirt3 deficiency also increased the luciferase activity of TCF/LEF reporter induced by TGF-β1, while Sirt3 overexpression decreased the luciferase activity in NRK-49F cells (Fig. 2h). Then, the promoter activity of downstream target genes was measured using ChIP assay. As shown in Fig. 2i, the promoter activity of twist was elevated after TGF-β1 stimulation. Sirt3 deficiency contributed to the promoter activity of twist, while Sirt3 overexpression suppressed twist promoter activity. The trends of snail1, MMP-7 and PAI-1 promoter activity in NRK-49F were similar to the trend of twist promoter activity. These results confirmed that Sirt3 deacetylated β-catenin and then suppressed following pro-fibrotic downstream target genes expression to reduce renal fibroblast activation and interstitial fibrosis.

### K49 site of β-catenin is mainly responsible for Sirt3-mediated β-catenin deacetylation

**Fig. 3:**
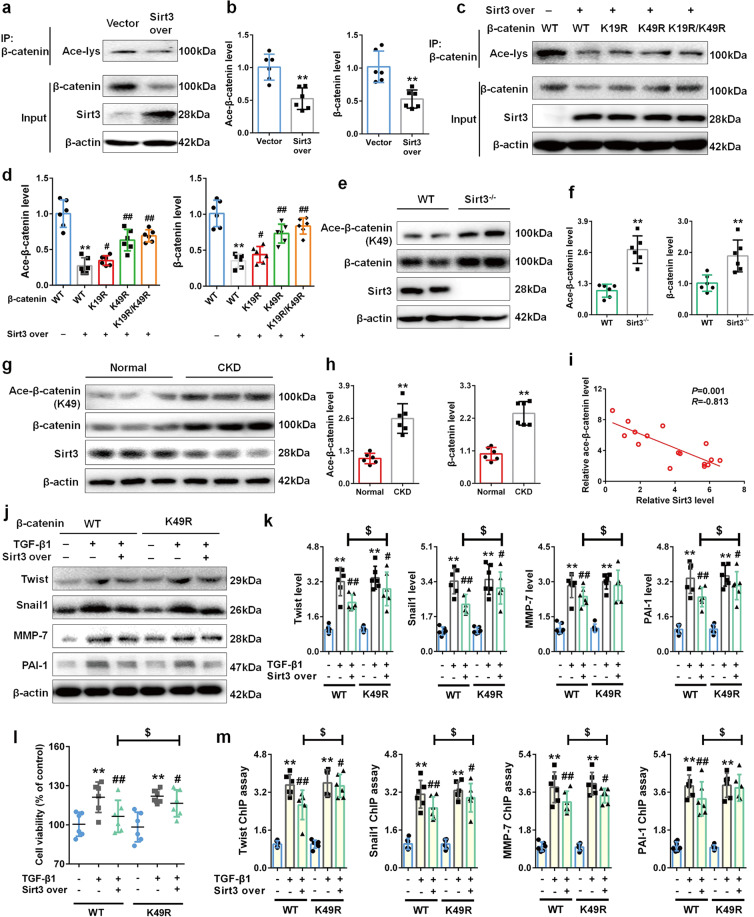
Sirt3 deacetylates β-catenin mainly via K49 site. **a**, **b** The protein expression and relative quantitative data of ace-β-catenin and β-catenin in NRK-49F cells after 48 h treatment. **c**, **d** The protein expression and relative quantitative data of ace-β-catenin and β-catenin in NRK-49F cells after 48 h treatment. **e**, **f** The protein expression and relative quantitative data of ace-β-catenin (K49) and β-catenin in WT and Sirt3^–/–^ mice. **g**, **h** The protein expression and relative quantitative data of ace-β-catenin (K49) and β-catenin in kidney tissues from patient with renal fibrosis. **i** Scatter plot for Sirt3 protein levels and ace-β-catenin (K49) protein levels. **j**, **k** The protein expression and relative quantitative data of twist, snail1, MMP-7 and PAI-1 in NRK-49F cells after 48 h treatment. **l** The cell viability of NRK-49F cells after 48 h treatment. **m** ChIP assay results of snail1, twist, MMP-7, and PAI-1 in NRK-49F cells after 48 h treatment. Data were presented as mean ± SD. Each dot presented the single data result in bar graph. ^**^P < 0.01 vs vector, WT or normal group (*n* = 6). ^#^*P* < 0.05, ^##^*P* < 0.01 vs TGF-β1, Sirt3^–/–^ or CKD group (*n* = 6). ^$^*P* < 0.05 vs TGF-β1 + Sirt3 over group (*n* = 6)

Further experiments focused on investigating the underlying mechanism of Sirt3-mediated β-catenin deacetylation. As shown in Fig. 3a, b, compared with vector group, overexpressing Sirt3 significantly inhibited the acetylated and total β-catenin protein levels in cultured NRK-49F cells, which confirmed that Sirt3 promoted β-catenin deacetylation and inhibited β-catenin protein expression. To fully address the underlying mechanisms of Sirt3 on β-catenin deacetylation and protein expression, we replaced the K19 and K49 by K19R and K49R that are acetylation-resistant arginine. As shown in Fig. 3c, d, co-IP results indicated that compared with WT β-catenin group, K19, K49R and K19R/K49R influenced Sirt3-mediated β-catenin deacetylation. Importantly, the expression of acetylated and total β-catenin in K49R group was higher than those in K19R group. The mutant of K49 into K49R significantly abolished Sirt3-mediated β-catenin deacetylation, indicating that K49 were mainly responsible for Sirt3-mediated β-catenin deacetylation, instead of K19. Consistently, compared with WT mice, Sirt3 deletion promoted β-catenin K49 acetylation and protein expression in vivo (Fig. 3e, f). We further detected the expression of Sirt3 and β-catenin in kidney tissues from patients with renal fibrosis. As shown in Fig. 3g, h, compared with normal group, the downregulation of Sirt3 protein and upregulation of acetylated β-catenin at K49 site were observed in CKD group. Notably, Sirt3 protein level was negatively corelated with the level of acetylated β-catenin at K49 site (Fig. 3i). These results from multiple species demonstrated that K49 site played an important role in Sirt3-mediated β-catenin deacetylation.

Further experiments investigated the importance of K49 in Sirt3-mediated β-catenin deacetylation during renal fibroblast activation. As shown in Fig. 3j, k, in the presence of WT β-catenin, Sirt3 overexpression significantly suppressed β-catenin downstream target genes expression induced by TGF-β1, while in the presence of K49R β-catenin, the inhibition of Sirt3 on downstream target genes was lowered. Cell viability and ChIP assay results showed a similar conclusion. Sirt3 overexpression significantly inhibited fibroblast viability, while the replacement of K49 into K49R significantly eliminated the effect of Sirt3 (Fig. 3l). As shown in Fig. 3m, Sirt3 overexpression suppressed the promoter activity of β-catenin downstream target genes, while the replacement of K49 into K49R facilitated their expressions. These results revealed that K49 site was mainly responsible for Sirt3-mediated β-catenin deacetylation to alleviate renal fibroblast activation and interstitial fibrosis, which firstly demonstrated that Sirt3 mediated β-catenin deacetylation mainly at K49 site.

### Sirt3-mediated β-catenin K49 deacetylation facilitates its ubiquitin-dependent degradation

**Fig. 4:**
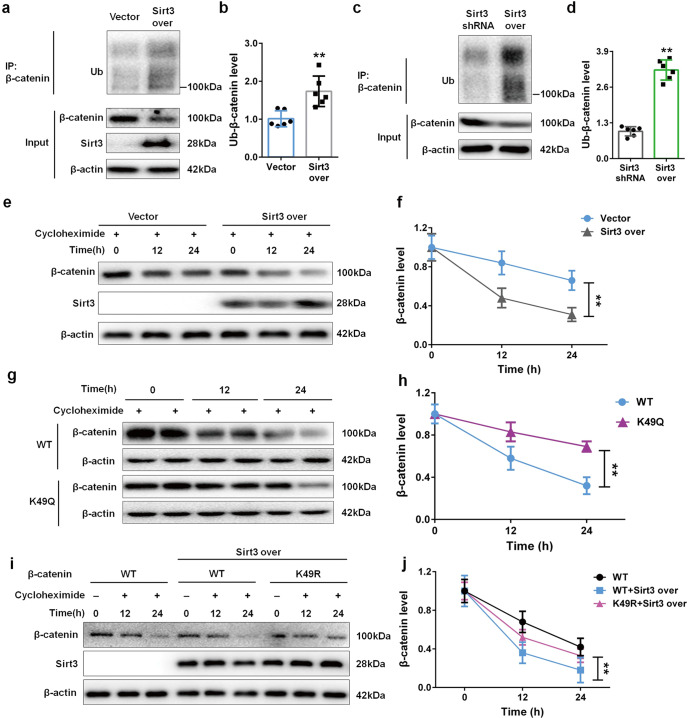
Sirt3 deacetylates β-catenin at K49 site and induces following ubiquitin-dependent degradation. **a**, **b** The protein expression and relative quantitative data of ubiquitin (Ub)-β-catenin in NRK-49F cells after 48 h treatment. **c**, **d** The protein expression and relative quantitative data of Ub-β-catenin in NRK-49F cells after 48 h treatment. **e**, **f** The protein expression and relative quantitative data of β-catenin in NRK-49F cells after 48 h treatment. **g**, **h** The protein expression and relative quantitative data of β-catenin in NRK-49F cells after 48 h treatment. **i**, **j** The protein expression and relative quantitative data of β-catenin in NRK-49F cells after 48 h treatment. Data were presented as mean ± SD. Each dot presented the single data result in bar graph. ^**^P < 0.01 vs vector, Sirt3 shRNA or WT group (*n* = 6).

Sirt3 not only induced β-catenin deacetylation also suppressed β-catenin protein, which suggested that Sirt3-mediated β-catenin deacetylation might interfere the ubiquitin-dependent degradation of β-catenin. Further experiments were designed to prove this. As shown in Fig. 4a, b, co-IP and Western blot results showed that compared with vector group, Sirt3 overexpression significantly promoted β-catenin ubiquitination and decreased total β-catenin expression in cultured NRK-49F cells. As shown in Fig. 4c, d, compared with Sirt3 over group, Sirt3 deficiency obviously suppressed the ubiquitination of β-catenin and maintained total β-catenin protein. The cycloheximide was used as a protein synthesis inhibitor. As shown in Fig. 4e, f, in the presence of cycloheximide, compared with vector group, Sirt3 overexpression significantly promoted β-catenin degradation in a time-dependent manner. These results indicated that Sirt3-mediated β-catenin deacetylation significantly affected its ubiquitin-dependent degradation.

To prove the role of K49 on Sirt3-mediated β-catenin deacetylation and following ubiquitin-dependent degradation, K49 mutated into K49Q that mimics acetylated β-catenin. As shown in Fig. 4g, h, compared with WT β-catenin group, K49Q delayed β-catenin degradation after treatment with cycloheximide for 12 h and 24 h. As shown in Fig. 4i, j, overexpressing Sirt3 accelerated β-catenin degradation in WT β-catenin group, while the presence of acetylation-insensitive K49R counteracted and weakened Sirt3 effect. These results demonstrated that Sirt3 mainly mediated β-catenin K49 deacetylation and then accelerated its ubiquitin-dependent degradation, resulting in the downregulation of pro-fibrotic downstream target gene expression.

### Pharmacological activation of Sirt3 by PAA mediates β-catenin K49 deacetylation to ameliorate renal fibroblast activation and interstitial fibrosis

**Fig. 5:**
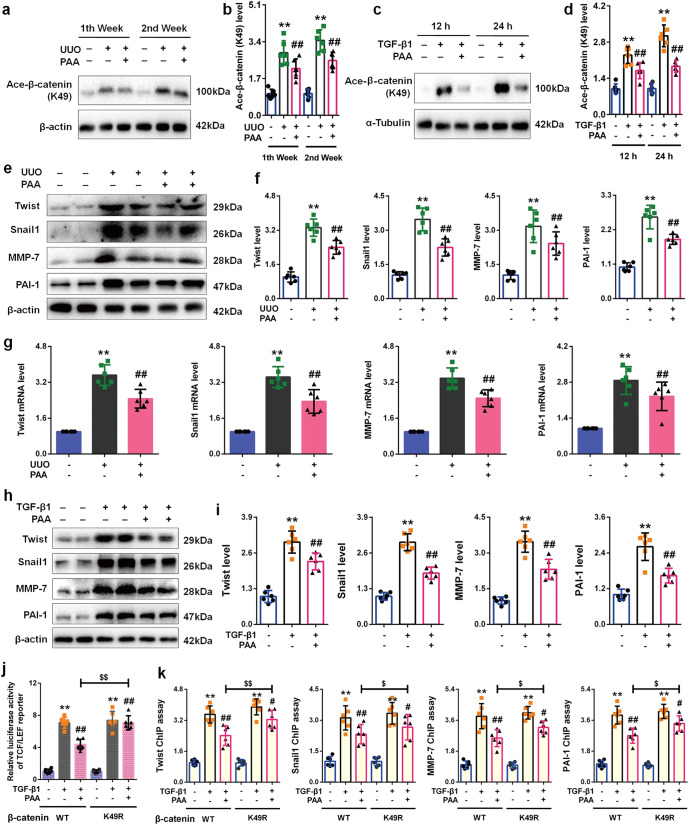
PAA activates Sirt3 to deacetylate K49 β-catenin thereby suppressing profibrotic downstream target gene expression. **a**, **b** The protein expression and relative quantitative data of ace-β-catenin (K49) in UUO rats. **c**, **d** The protein expression and relative quantitative data of ace-β-catenin (K49) in NRK-49F cells. **e**, **f** The protein expression and relative quantitative data of twist, snail1, MMP-7 and PAI-1 in UUO rats at 2nd week. **g** The mRNA expression of twist, snail1, MMP-7 and PAI-1 in UUO rats at 2nd week. **h**, **i** The protein expression and relative quantitative data of twist, snail1, MMP-7 and PAI-1 in NRK-49F cells after 24 h treatment. **j** The luciferase activity of TCF/LEF reporter in NRK-49F cells after 24 h treatment. **k** ChIP assay results of snail1, twist, MMP-7, and PAI-1 in NRK-49F cells after 24 h treatment. Data were presented as mean ± SD. Each dot presented the single data result in bar graph. ^**^P < 0.01 vs sham-operated, control or WT group (*n* = 6). ^*#*^*P* < 0.05, ^##^*P* < 0.01 vs UUO, TGF-β1 or K49R group (*n* = 6). ^$^*P* < 0.05, ^$$^*P* < 0.01 *vs* TGF-β1 + Sirt3 over group (*n* = 6)

Further experiments elucidated the effect of pharmacological activation of Sirt3 by PAA on β-catenin. As shown in Fig. 5a, b, pharmacological activation of Sirt3 by PAA significantly alleviated β-catenin K49 acetylation in UUO rats at 1st and 2nd weeks. Similar results were observed in cultured NRK-49F cell. PAA treatment obviously suppressed β-catenin K49 acetylation (Fig. 5c, d). These data confirmed that PAA activated Sirt3 to deacetylate β-catenin K49 against renal fibroblast activation and interstitial fibrosis. We further investigated whether pharmacological activation of Sirt3 by PAA reduced β-catenin downstream target gene expressions. As shown in Fig. 5e–g, the protein and mRNA expressions of twist, snail1, MMP-7, and PAI-1 were obviously upregulated in UUO group, and downregulated after PAA treatment. In cultured NRK-49F cells, the protein expression of twist, snail1, MMP-7, and PAI-1 was elevated after TGF-β1 stimulation, but reduced by PAA treatment (Fig. 5h, i). These data confirmed that pharmacological activation of Sirt3 by PAA suppressed β-catenin K49 acetylation and following pro-fibrotic β-catenin downstream target gene expressions thus ameliorating renal fibroblast activation and interstitial fibrosis.

We conducted additional experiments to investigate the role of K49 β-catenin in pharmacological activation of Sirt3 by PAA. As shown in Fig. 5j, PAA treatment significantly suppressed the luciferase activity of TCF/LEF reporter, while the replacement of K49 into K49R obviously counteracted the effect of PAA in NRK-49F cells. The results of ChIP assay confirmed the conclusion. In the presence of K49R, the inhibitory effect of PAA on the promoter activity of downstream target genes was lowered than those in WT β-catenin group (Fig. 5k), indicating that PAA exerted anti-fibrotic effect mainly via Sirt3-mediated β-catenin K49 deacetylation. Taken together, pharmacological activation of Sirt3 by PAA reduced renal fibroblast activation and interstitial fibrosis by lowering β-catenin downstream target gene expression mainly via Sirt3-mediated β-catenin K49 deacetylation.

### Sirt3 is essential for PAA to attenuate renal fibroblast activation and interstitial fibrosis

**Fig. 6:**
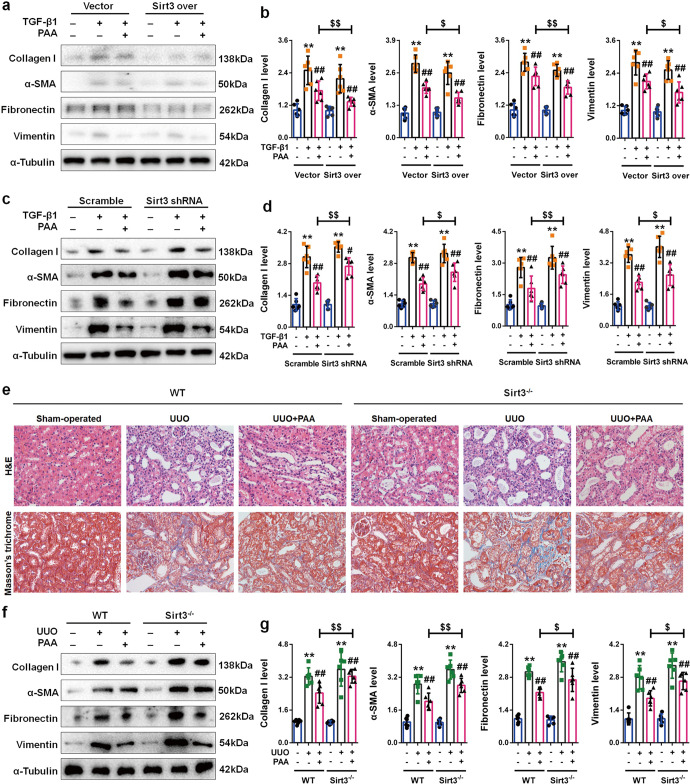
Sirt3 is required for PAA to alleviate renal fibroblast activation and interstitial fibrosis. **a**, **b** The protein expression and relative quantitative data of collagen I, α-SMA, fibronectin and vimentin in NRK-49F cell after 48 h treatment. **c**, **d** The protein expression and relative quantitative data of collagen I, α-SMA, fibronectin and vimentin in NRK-49F cell after 48 h treatment. **e** H&E and Masson’s trichrome stainings of kidney tissues in WT and Sirt3^–/–^ UUO mice at 1st week. Magnification, ×400. **f**, **g** The protein expression and relative quantitative data of collagen I, α-SMA, fibronectin and vimentin in WT and Sirt3^–/–^ UUO mice at 1st week. Data were presented as mean ± SD. Each dot presented the single data result in bar graph. ^**^P < 0.01 vs vector, scramble or sham-operated group (*n* = 6). ^#^*P* < 0.05, ^##^*P* < 0.01 *vs* TGF-β1 or UUO group (*n* = 6). ^$^*P* < 0.05, ^$$^*P* < 0.01 vs TGF-β1 + PAA or UUO + PAA group (*n* = 6)

For a rigorous characterization of Sirt3 role in the anti-fibrotic effect of PAA, we used lentivirus expressing Sirt3 over and Sirt3 shRNA. As shown in Fig. 6a, b, in vector-transfected group, PAA significantly reduced the upregulation of collagen I, α-SMA, fibronectin, and vimentin protein after TGF-β1 stimulation, while Sirt3 overexpression enhanced the anti-fibrotic effect of PAA, indicated by the downregulation of collagen I, α-SMA, fibronectin, and vimentin protein in Sirt3 over-transfected TGF-β1 + PAA group. On the contrary, the protein expression of collagen I, α-SMA, fibronectin, and vimentin in Sirt3 shRNA-transfected TGF-β1 + PAA group were higher than those in scramble-transfected TGF-β1 + PAA group, indicating Sirt3 deficiency weakened the anti-fibrotic effect of PAA (Fig. 6c, d). These data revealed that PAA alleviated renal fibroblast activation in a Sirt3-dependent manner.

**Fig. 7:**
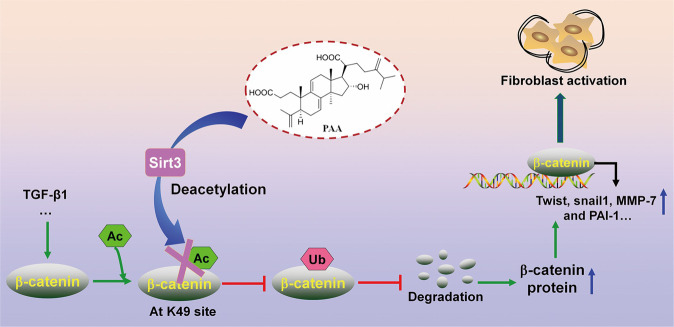
Sirt3 was required for PAA to reduce renal fibroblast activation and interstitial fibrosis via enhancing β-catenin K49 deacetylation. Sirt3 functions as the therapeutic target of PAA to deacetylate β-catenin at K49 site thus suppressing pro-fibrotic downstream target gene expression to alleviate renal fibroblast activation and interstitial fibrosis. During renal fibrosis, the stimulation of pro-fibrotic factors, such as TGF-β1, induced β-catenin acetylation and prevented β-catenin degradation, resulting in the upregulation of pro-fibrotic downstream target genes, and eventually caused renal fibroblast activation and interstitial fibrosis. Sirt3 specifically mediated the deacetylation of β-catenin at K49 site, thereby promoting β-catenin ubiquitin-dependent degradation. PAA activated Sirt3 to exhibit the inhibitory effects on renal fibroblast activation and interstitial fibrosis, and the effects of PAA was in a Sirt3-dependent manner

To draw a convincing conclusion that Sirt3 was required for the anti-fibrotic effect of PAA, WT and Sirt3^-/-^ UUO mice were employed. H&E and Masson’s trichrome staining results elucidated that compared with WT UUO + PAA group, Sirt3 deletion eliminated the anti-fibrosis of PAA and aggravated renal interstitial fibrosis at 1th week after UUO surgery (Fig. 6e). Moreover, compared with WT UUO + PAA group, Sirt3 deletion also promoted the upregulation of collagen I, α-SMA, fibronectin, and vimentin in UUO + PAA group (Fig. 6f, g). These results supported the conclusion that Sirt3 was required for PAA to attenuate renal fibroblast activation and interstitial fibrosis. In summary, PAA exhibited anti-fibrotic effect in a Sirt3-dependent manner, and Sirt3 was required for PAA to attenuate renal fibroblast activation and interstitial fibrosis by mediating β-catenin K49 deacetylation (Fig. 7).

## Discussion

In the present study, we reveal that PAA modulates Sirt3 to attenuate renal fibroblast activation and interstitial fibrosis by mediating β-catenin K49 deacetylation. Firstly, molecular docking analysis and experiment results shows the possible interaction between Sirt3 and PAA, and pharmacological activation of Sirt3 by PAA during renal fibroblast activation and interstitial fibrosis. Furthermore, we firstly demonstrate that Sirt3 attenuates renal fibroblast activation and interstitial fibrosis via mediating β-catenin K49 deacetylation. Functionally, Sirt3 specifically deacetylates β-catenin at K49 site, accelerates following ubiquitin-dependent degradation, and suppresses pro-fibrotic downstream target gene expression. PAA activates Sirt3 to induce β-catenin K49 deacetylation thus reducing pro-fibrotic downstream target gene expression. Our results in this study underscore that the inhibitory effect of PAA on renal fibroblast activation and interstitial fibrosis is in a Sirt3-dependent manner, and Sirt3 is required for the anti-fibrosis of PAA, which provide the promising therapeutic target and strategy to attenuate renal fibroblast activation and interstitial fibrosis.

Renal interstitial fibrosis is increasingly recognized as a dominant reason for morbidity and mortality of CKD. It has been for a long time that fibrosis was thought to be relentlessly irreversible and progressive. However, there is a great deal of research indicating that the progression of fibrosis, a highly dynamic process, can be substantially disturbed by therapeutic interventions [26]. The inhibition of fibroblast activation is considered as the potential therapeutic strategy to delay fibrosis since it specifically targets the pathogenesis of fibrosis. The activated fibroblast is the main effect cell of renal interstitial fibrosis, which triggers ECM remodeling and epithelial-mesenchymal transition (EMT) after chronic injury [4]. Emerging evidence confirms that the inhibition of fibroblast activation functions as an effective strategy to decay renal interstitial fibrosis progression and kidney function decline [27, 28]. In this study, we employed UUO model and TGF-β1-stimulated NRK-49F cell to prove the effect of PAA against renal fibroblast activation and interstitial fibrosis, and both molecular docking analysis and experiment results indicated that PAA increased Sirt3 expression and deacetylase activity.

Sirt3 belongs to Sirtuin family that is a group of NAD^+^-dependent deacetylases. Sirt3 is reported to alleviate renal fibrosis [13, 29, 30]. Sirt3 modulates the deacetylation of several pro-fibrotic or anti-fibrotic factors to suppress fibrosis, including p53, PRDX3, and SENP1 [31–33]. However, the inhibitory effect role of Sirt3 on fibroblast activation in kidney has been hardly reported, and no evidences have been indicated the regulation of Sirt3 on β-catenin deacetylation. Here, we first identified Sirt3 significantly suppressed renal fibroblast activation and interstitial fibrosis and explored its mechanism. Sirt3 significantly decreased in fibrotic kidneys from UUO models and patients with renal fibrosis. In cultured NRK-49F cells, Sirt3 deficiency facilitated TGF-β1-stimulated renal fibroblast activation, while Sirt3 overexpression significantly reduced fibroblast activation. Using Sirt3^-/-^ mice, we confirmed that Sirt3 deletion accelerated renal injury and interstitial fibrosis after UUO surgery. These data revealed the anti-fibrotic effect of Sirt3 in kidney. Pharmacological activation of Sirt3 by PAA significantly reduced renal fibroblast activation and interstitial fibrosis, indicating PAA activates Sirt3 against renal fibrosis. Notably, PAA exerted the inhibitory effect on renal fibroblast activation and interstitial fibrosis in a Sirt3-dependent manner, and Sirt3 was required for PAA, highlighting Sirt3 functioned as a promising therapeutic target for renal fibrosis.

The most important finding of the present study is identifying a previously unrecognized role of Sirt3 on β-catenin deacetylation. β-Catenin plays a definitely significant role in renal fibroblast activation and interstitial fibrosis. Whereas the importance of β-catenin phosphorylation via modulating casein kinase 1α and glycogen synthase kinase-3β in controlling β-catenin stability is well established [16, 34, 35], the potential role of acetylation/deacetylation in β-catenin stability has been largely overlooked in the field. Here, we provided a novel discovery that Sirt3 induced β-catenin K49 deacetylation and following ubiquitin-dependent degradation, which a new strategy to regulate β-catenin activity. Notably, Sirt3 did not affect β-catenin mRNA expression, but only affected its deacetylation and then promoted following ubiquitin-dependent degradation. Sirt3 overexpression reduced β-catenin acetylation and facilitated ubiquitin-dependent degradation, while Sirt3 deficiency promoted β-catenin acetylation and suppressed degradation to evoke fibroblast activation. The negative correlation of Sirt3 and acetylated β-catenin levels were also observed in clinical kidney tissues. However, the number of kidney tissues used in this study were limited, and larger amounts of samples were needed to further confirm this conclusion. Additionally, pharmacological activation of Sirt3 by PAA promoted β-catenin deacetylation and then reduced pro-fibrotic downstream target gene expression to inhibit renal fibroblast activation and interstitial fibrosis.

One of the novel and interesting findings in this study is that the identification of β-catenin K49 deacetylation is vital for Sirt3 against fibrosis. The acetylation occurs at lysine residues, and lysine residues in β-catenin are lysine-11, K19, K49, and lysine-394. Considering that the N-terminal 1–49 amino acids determines the stability of β-catenin and K49 and K19 can be ubiquitinated and/or acetylated [9], we mainly focused on K19 and K49 sites here. The replacement of K19 into acetylation-resistant K19R in β-catenin hardly disturbed the deacetylase activity of Sirt3, while the replacement of K49 into K49R significantly vanished Sirt3 deacetylase activity in NRK-49F cells, suggesting that K49 was mainly responsible for β-catenin deacetylation by Sirt3. Furthermore, K49Q that functions as pseudo-acetylated K49-β-catenin prevented β-catenin degradation, while acetylation-resistant K49R vanished β-catenin deacetylation induced by Sirt3, indicating the important role of β-catenin K49 deacetylation in its ubiquitin-dependent degradation which is consistent with previous studies that emphasize the importance of K49 [11, 36, 37]. These data elucidated Sirt3 induced β-catenin K49 deacetylation and degradation to suppress renal fibroblast activation and interstitial fibrosis. Notably, PAA activated Sirt3 and then induced β-catenin deacetylation at K49 site, thereby reducing pro-fibrotic downstream target gene expressions. To the best of our knowledge, this study is the first report that Sirt3 deacetylates β-catenin at K49 site to suppress renal fibroblast activation.

β-Catenin leads to renal fibroblast activation and interstitial fibrosis through mediating pro-fibrotic downstream target gene expression, especially twist, snail1, MMP-7, and PAI-1. Twist and snail1 directly drive fibrogenesis and are the key transcription factor to trigger EMT and ECM deposition [38, 39]. MMP-7 is not only a biomarker of renal interstitial fibrosis [40], but also a key mediator to suppress ECM degradation [8, 22]. PAI-1 promotes ECM deposition, while deletion of PAI-1 ameliorates renal interstitial fibrosis [41]. In the present study, ChIP assays results showed that Sirt3 overexpression significantly inhibited the protein expression and promoter activity of twist, snail, MMP-7, and PAI-1 via deacetylating K49 β-catenin. After the replacement of K49 into K49R, pharmacological activation of Sirt3 by PAA exhibited the weakened inhibitory effects on twist, snail, MMP-7, PAI-1 expression and promoter activity, indicating that β-catenin K49 deacetylation mediated by Sirt3 was vital for PAA effect.

Taken together, we herein report that PAA modulates Sirt3 to mediate β-catenin K49 deacetylation against renal fibroblast activation and interstitial fibrosis. It is firstly elucidated that Sirt3 induces β-catenin deacetylation and following ubiquitin-dependent degradation mainly via K49 site, thus suppressing pro-fibrotic downstream target gene expressions. PAA exerts anti-fibrotic effect in a Sirt3-dependent manner, and Sirt3 is required for PAA against renal fibroblast activation and interstitial fibrosis. Our finding also highlights that Sirt3 functions as a promising therapeutic target against renal fibroblast activation and interstitial fibrosis.

## Supplementary information

Supplementary materials
