# Supplementary material for: Poricoic acid A suppresses renal fibroblast activation and interstitial fibrosis in UUO rats via upregulating Sirt3 and promoting β-catenin K49 deacetylation
Source: Acta Pharmacol Sin. 2022 Dec 5;44(5):1038–50. doi: 10.1038/s41401-022-01026-x (PMC10104829; doi:10.1038/s41401-022-01026-x)
Supplement: Supplementary file 1 — Supplementary materials [file 41401_2022_1026_MOESM1_ESM.docx]

**Supplementary materials**

**
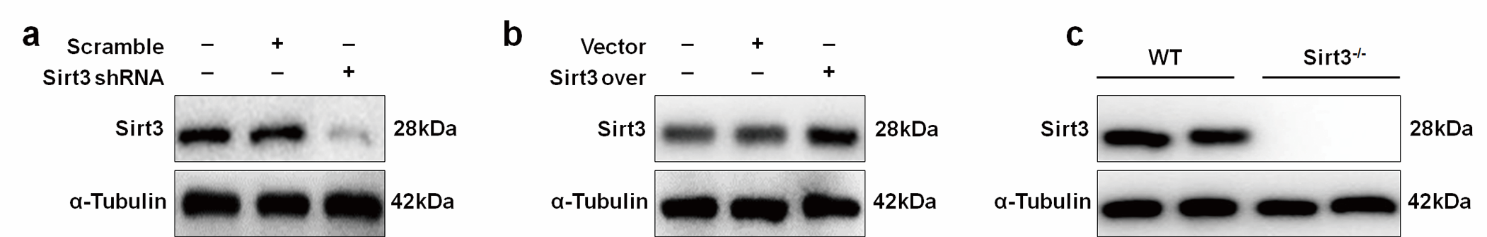
**

**Fig S1. The Sirt3 protein expression in NRK-49F cells and mice.** (a) The Sirt3 protein expression in NRK-49F after transfection with Sirt3 shRNA or scramble at 48 h. (b) The Sirt3 protein expression in NRK-49F after transfection with Sirt3 over or vector at 48 h. (c) The Sirt3 protein expression in WT and Sirt3^-/-^ mice.


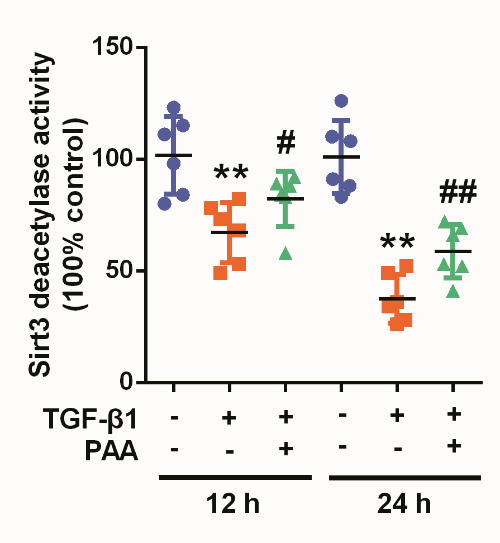


**Fig S2. The Sirt3 deacetylase activity in NRK-49F cells after 12 h and 24 h treatment.**

**Table S1. The information of primary antibodies**

| **Primary antibody** | **Catalog number** | **Manufacturer** | **Dilution ratio** |
| --- | --- | --- | --- |
| Acetylated-lysine | 9441 | Cell Signaling Technology | 1:2000 |
| β-catenin | 610154 | BD Transduction Laboratories | 1:1000 |
| Sirt3 | ab189860 | Abcam | 1:500 |
| Ace-β-catenin (K49) | 9030 | Cell Signaling Technology | 1:2000 |
| Ub | sc-8017 | Santa Cruze | 1:1000 |
| Twist | ab50887 | Abcam | 1:1000 |
| Snail1 | 3879 | Cell Signaling Technology | 1:2000 |
| MMP-7 | PA5-87486 | Invitrogen | 1:1000 |
| PAI-1 | 11907 | Cell Signaling Technology | 1:2000 |
| Collagen I | 91144 | Cell Signaling Technology | 1:2000 |
| α-SMA | 19245 | Cell Signaling Technology | 1:2000 |
| Fibronectin | ab2413 | Abcam | 1:1000 |
| Vimentin | ab92547 | Abcam | 1:1000 |
| β-actin | 20536-1-AP | Proteintech | 1:2000 |

**Table S2. The *Rattus norve* primers used for qPCR and ChIP**

| **Gene** | **Forward (5’-3’)** | **Reverse (5’-3’)** | **Product size (bp)** |
| --- | --- | --- | --- |
| qPCR | | | |
| Sirt3 | CTGAGCTGTCGACTGGTATTG | TGTGTCCTCCACATCCAAAG | 112 |
| β-catenin | CTCAGATGGTGTCTGCCATAG | TGGTGGGAAAGGTTGTGTAG | 97 |
| Twist | AGCTGAGCAAGATTCAGACC | AGCTTGCCATCTTGGAGTC | 99 |
| Snail1 | CCACTCGGATGTGAAGAGATAC | AGACTCTTGGTGTTTGTGGAG | 85 |
| MMP-7 | GACTTGCCTCGGTTCTTAGTAG | CCCTTGCGAAGCCAATTATG | 127 |
| PAI-1 | CTGGTGAACGCCCTCTATTT | ATGGTGCTACCATCAGACTTG | 98 |
| β-actin | ACAGGATGCAGAAGGAGATTAC | ACAGTGAGGCCAGGATAGA | 117 |
| ChIP | | | |
| Twist | ACCTCTTTGAAGCTCTTGGG | GGAGATCGCGTGAGTAGTTATG | 109 |
| Snail1 | CGCTACCGTAGATGGGAATTAG | ACTTCATTACTGCTGGGTGTAG | 130 |
| MMP-7 | TCTCTCTCTCTCTCTCTCTCTCT | GCGAGAGGCCTTAGTTTCTATT | 100 |
| PAI-1 | CAAGGTGCAGGTCTTGAATTG | CTCCTCTCCCTTCTTCCTTCTA | 102 |
